# Are the Global Initiative for Asthma (GINA) Guidelines Being Correctly Used to Diagnose Severe Asthma in the UAE?

**DOI:** 10.7759/cureus.12278

**Published:** 2020-12-25

**Authors:** Mohamed Abuzakouk, Sonya Jacob, Omar Ghorab

**Affiliations:** 1 Allergy and Immunology, Cleveland Clinic Abu Dhabi, Abu Dhabi, ARE

**Keywords:** asthma, gina guidelines, severe asthma, clinical audit

## Abstract

Objectives

We aimed to identify the percentage inaccuracy in classifying asthma severity as severe asthma based on the 2019 Global Initiative for Asthma (GINA) guidelines criteria, at Cleveland Clinic Abu Dhabi, and make recommendations to improve the assessment of asthma severity.

Methods

All asthma patients that attended the Pulmonology clinic or the Allergy clinic from May 2015 to December 2019 were retrospectively analyzed to identify which asthma patients classified as having severe asthma according to the 2019 GINA guidelines criteria. We then calculated the percentage inaccuracy associated with giving diagnoses of severe asthma.

Results

We retrospectively analyzed a total of 902 patients, and out of those, we identified 334 as patients with severe asthma according to the 2019 GINA guidelines criteria. Of those 334 patients, 218 were given an incorrect asthma severity of either mild (N=14), moderate (N=203), or unspecified asthma severity (N=1) in the hospital’s electronic records. This represents a percentage inaccuracy of 65.3% in classifying asthma severity as severe asthma. Fluticasone propionate-salmeterol was the most used ICS-LABA (inhaled corticosteroid and long-acting beta-agonist) medication in the severe asthma group (58.1%). Fluticasone furoate-vilanterol was identified as the most incorrectly prescribed ICS-LABA medication (68.2%).

Conclusion

We identified an inaccuracy of 65.3% in classifying asthma severity as severe at our hospital. This inaccuracy is associated with a lack of understanding of the GINA guidelines by clinicians, as well as a lack of acceptance of some of the criteria in the GINA guidelines by patients. We have made recommendations to help improve the accuracy of asthma severity assessment, in order to be fully adherent to the GINA guidelines criteria.

## Introduction

This article was previously presented as a conference abstract at the 2020 EAACI Congress on June 6, 2020, and the 2020 ERS International Congress on September 5, 2020. Asthma is a chronic lung disease that is estimated to affect 300 million people worldwide [1]. Moreover, it presents major health, social and economic burdens for patients [2]. Asthma is the 16th leading cause of years lived with disability and the 28th leading cause of burden of disease, measured by disability-adjusted life years [1]. In the United Arab Emirates (UAE), asthma has been reported to affect almost 4.9% of the total population [3].

The Global Initiative for Asthma (GINA) is a medical organization that was established to increase asthma awareness among the public, and to help improve the prevention and management of asthma, via working with healthcare professionals and public health officials worldwide [4]. In 2019, GINA published their latest guidelines [4], which included updates on the treatment of mild asthma. It included the most important change in asthma management in 30 years, which was that all asthma patients must not be placed on short-acting beta2-agonists alone, but they must also receive daily dose inhaled corticosteroid (ICS), to reduce the frequency of exacerbations. Furthermore, GINA recommends that asthma should be classified retrospectively based on the controller medication ICS-LABA (inhaled corticosteroid-long acting beta-agonist) that the patient has been on for a number of months. GINA classifies asthma severity as: 1) Mild asthma, which can be treated with daily/as-needed low dose ICS-LABA with optional leukotriene receptor antagonist (LTRA), 2) Moderate asthma, which is treated with daily low or medium dose ICS-LABA with optional LTRA, and 3) Severe asthma, which is treated with daily high-dose ICS-LABA with optional tiotropium or biological therapy. Additionally, GINA dictates that in order for a patient to be diagnosed with severe asthma, they must: 1) Be compliant with their daily medication, 2) Practice good inhaler technique, and 3) Be treated for risk factors that may contribute to asthma presentation. If these conditions are not met, the patient is said to have uncontrolled asthma. GINA recommends that a patient is started on a specific medication type and dosage based on the clinical presentation of the patient when they are first seen, and then upon follow-up after a few months, the patient’s medication dose should either be stepped up if they present with frequent symptoms and exacerbations or stepped down if they present with few symptoms.

The correct identification of asthma severity is important in making clinical decisions regarding the treatment modalities of asthma. From a patient’s perspective, being on long-term high-dose ICS-LABA is associated with an increased risk of local and systemic side effects [5]. From a hospital’s perspective, the incorrect classification of asthma severity in hospital records could potentially lead to major financial losses. We have, therefore, carried out a clinical audit to investigate the accuracy of classifying asthma severity as ‘Severe Asthma’ in the Allergy & Immunology Clinic and Pulmonology Clinic, at Cleveland Clinic Abu Dhabi, a large tertiary hospital in the United Arab Emirates.

## Materials and methods

We pooled a total of 1436 asthma patients who attended the hospital from May 2015 to December 2019. We excluded patients who were deceased (N=4), and patients with asthma-chronic obstructive pulmonary disease (COPD) overlap syndrome (N=6). We retrospectively classified the cohort into five groups. Patients were classified into one of the groups based on the 2019 GINA guidelines criteria. Additionally, patients must have completed at least three consecutive visits to the Allergy clinic or Pulmonology clinic at our hospital, with each visit being at least one month apart from one another. Where patients completed more than three visits to the clinic, the last three visits were considered in our analysis. Patients that are on biological therapy for asthma with a history of high-dose ICS prior to being put on biological therapy were considered in our cohort. Relating to the hospital’s electronic records system (EPIC), where there was a discrepancy in the medication type or dosage between the pharmacy report and the doctor’s report, the pharmacy’s report was considered for this analysis. Data on compliance were collected from the clinicians' notes. A total of 902 patients fulfilled our study criteria, of whom 334 were severe asthma patients and were used for our analysis.

## Results

**Figure 1:**
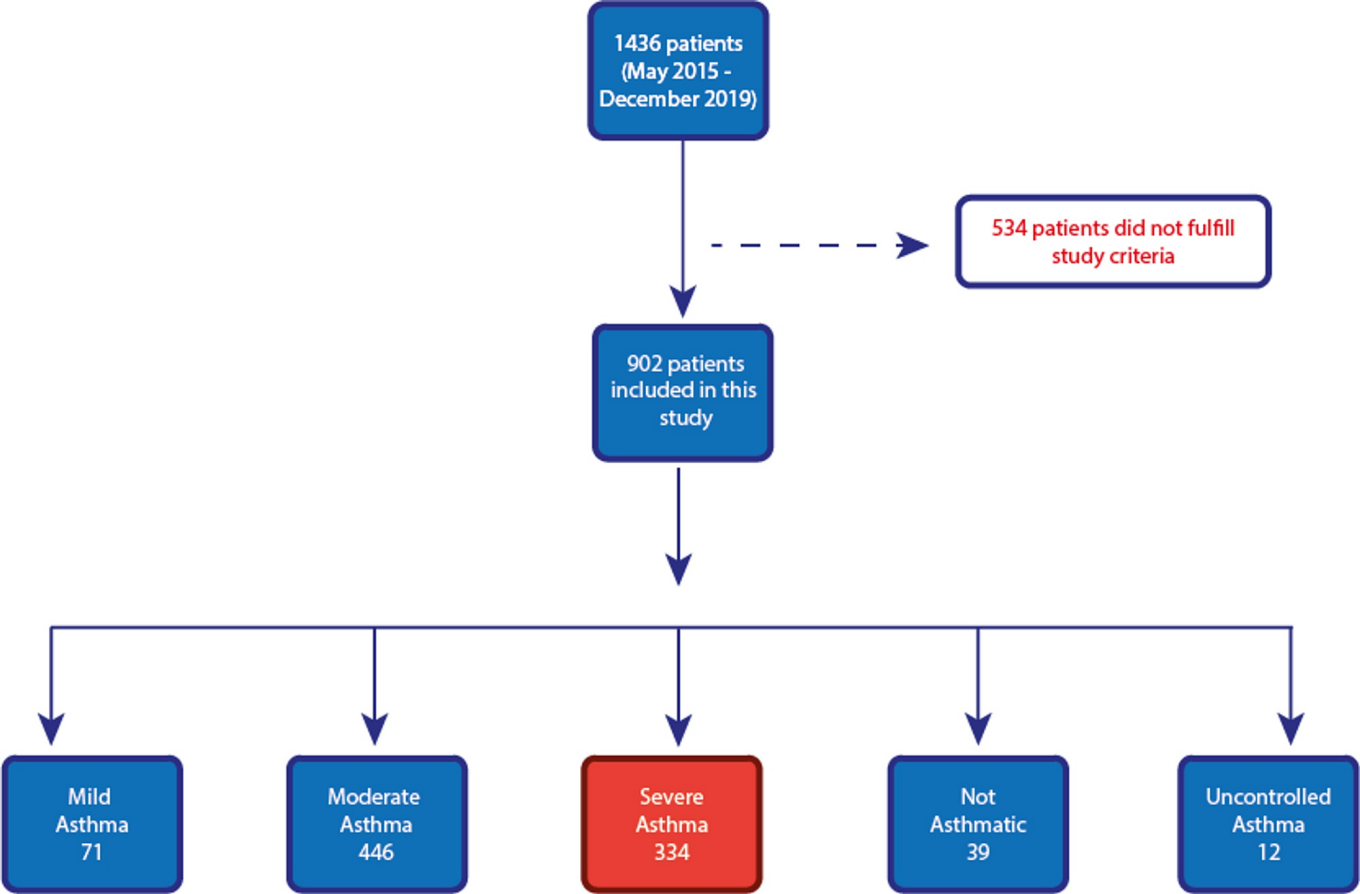
Detailed breakdown of the total cohort

The total cohort (N=902) was analyzed and divided into five groups: 1) mild asthma (N=71), 2) moderate asthma (N=446), 3) severe asthma (N=334), 4) not asthmatic (N=39), and 5) uncontrolled asthma (N=12)(Figure 1). All patients in the ‘uncontrolled asthma’ group were not compliant with their medication. All 39 patients in the ‘non-asthmatic group’ were given a diagnosis of asthma on their first visit to the hospital, however, upon subsequent visits and diagnostic procedures, they were given different diagnoses, most commonly bronchiectasis and cystic fibrosis.

Three-hundred and thirty-four out of the 902 patients were classified as severe asthmatics according to the 2019 GINA guidelines. Of those 334 patients, 218 have been incorrectly classified in the hospital’s electronic records system - more specifically, 203 as moderate asthma, 14 as mild asthma, and as unspecified asthma severity. This represents a percentage inaccuracy of 65.3%.

We divided patients in the severe asthma category (N=334) into groups based on the daily ICS-LABA medication they were on (Table 1). The most commonly used ICS-LABA was fluticasone propionate-salmeterol (N=194), followed by budesonide-formoterol (N=96) and fluticasone furoate-vilanterol (N=44).

**Table 1:** Breakdown of each ICS-LABA medication group ICS-LABA: inhaled corticosteroid and long-acting beta-agonist

ICS-LABA	Total number of patients on severe asthma dosage	Number of patients on severe asthma dosage, but given incorrect asthma severity	% inaccuracy
Fluticasone propionate-salmeterol	194	132	68.0
Fluticasone furoate-vilanterol	44	30	68.2
Budesonide-formoterol	96	56	58.3

**Figure 2:**
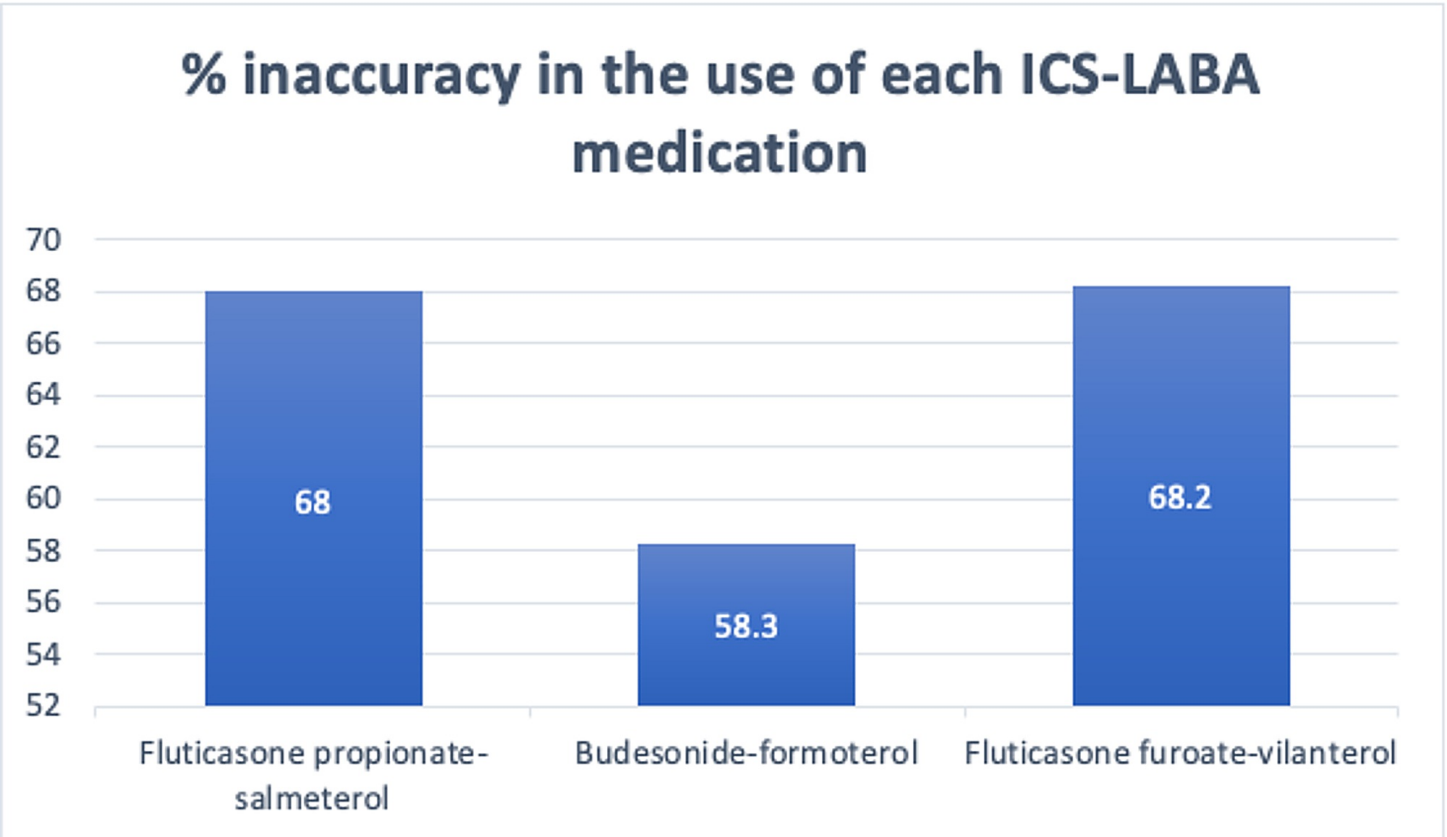
Percentage inaccuracy in the use of each ICS-LABA medication ICS-LABA: inhaled corticosteroid and long-acting beta-agonist

We further analyzed these groups to identify the number of patients on medication dosage that classifies them as having severe asthma, but were given a lower asthma severity in the hospital records, and therefore identify the ICS-LABA medication that is misunderstood the most (Figure 2). The results showed that fluticasone furoate-vilanterol was the most incorrectly prescribed ICS-LABA medication (68.2%), followed by fluticasone propionate-salmeterol (68.0%) and budesonide-formoterol (58.3%).

## Discussion

A 65.3% inaccuracy in classifying asthma severity as severe asthma at the hospital is deemed to be a high percentage. We assume that such a high percentage inaccuracy was to the detriment of the hospital from a financial point of view, as many patients with severe asthma were incorrectly classified as other asthma severities and, therefore, must have been given a lower clinic visit valuation.

When we discussed these findings with our clinicians, many stated that the majority of patients preferred not being stepped down on their ICS-LABA medication when their disease state had been stable for a long period of time and, as a result, clinicians were not able to correctly classify asthma severity according to the GINA guidelines criteria. More importantly, the patients put themselves at risk for the long-term adverse effects of inhaled corticosteroids, which have been studied thoroughly and shown to specifically have an effect on the hypothalamus-pituitary-adrenal axis, bone density, growth, eyes, skin, as well as the immune system, leading to an increased risk of pneumonia [5]. Therefore, our first action plan was to ask clinicians to provide patients with detailed insight on the systemic side effects of high-dose, long-term inhaled corticosteroids and, therefore, help increase their uptake of the stepping down approach for medication that is recommended by the GINA guidelines criteria.

In our retrospective analysis, we observed, according to clinicians' notes, that all the patients were symptomatically stable on their medications for at least three months, and, therefore, we were led to believe that there is a degree of inaccuracy associated with patient diagnoses, rather than with their prescriptions. This was similarly observed in two other studies on the adherence of clinicians to the GINA guidelines criteria; Nguyen et al. [6] and Umoh et al. [7] showed that only 22% and 36% of their clinicians treating asthma patients had a correct understanding of the GINA guidelines that was applied to treatment decisions. Therefore, we have started to educate all clinicians treating asthma patients of the GINA guidelines via educational programs such as hospital grand rounds and departmental meetings. Furthermore, there seems to be a misunderstanding of the dosage charts of the specific ICS-LABA medications, especially of fluticasone propionate-salmeterol, as shown in Figure 2. Therefore, clarifications with regards to the dosages of these medications will be made for all clinicians treating patients with asthma.

Another element to be considered is the hospital’s electronic records system, which proved to be unreliable for obtaining accurate data that could be utilized in studies and analyses. In collaboration with the hospital’s IT department, we put forward suggestions to facilitate a more accurate recording of data by clinicians. One suggestion was to register all the doses of the different ICS-LABA medications and directly link them to the different asthma severities based on the GINA guidelines criteria so that the clinician would only have to record the medication that the patient is on, and the system would automatically record the correct asthma severity. The system would prevent clinicians from being able to close the patient file without registering the correct asthma severity that corresponds with the ICS-LABA medication prescribed and, therefore, it prompts clinicians to choose the correct asthma severity.

Assessment of clinician understanding and application of the GINA guidelines criteria for successful treatment decisions in our retrospective study was carried out by comparing the assigned asthma severity for each patient with the ICS-LABA medication they were on. In order to directly assess clinician understanding and application of the GINA guidelines criteria, we could have devised questionnaires that tested clinician understanding of the different aspects of the GINA guidelines criteria, including asthma diagnosis, severity assessment, and risk factor management. Additionally, another way the adherence with GINA guidelines criteria could have been assessed was via checking whether patients were being correctly stepped up or stepped down on treatment, using comparisons before and after the treatment of objective measures such as the asthma control test (ACT) or pulmonary function test (PFT).

## Conclusions

We identified an inaccuracy of 65.3% in classifying asthma severity as severe at our hospital. The two main goals that the GINA guidelines criteria include risk reduction (asthma-related death, exacerbations, airway damage, and medication side effects) and symptom control. We have identified that there is a lack of awareness and understanding of the GINA guidelines by clinicians. Therefore, the main action plan is to educate clinicians on the correct use of the GINA guidelines and patients of the implications of not following what has been specified by the GINA guidelines criteria. Asthma is a chronic disease that requires a successful partnership between the patient and clinician in order to achieve the most effective management plans, and a correct understanding of the GINA guidelines criteria promises to achieve that. Moreover, further studies should be conducted to understand the reasons behind the lack of acceptance and practice of the GINA guidelines criteria by many clinicians. Perhaps, GINA could produce a simpler version of their guidelines, and in specific of the ICS-LABA dosing charts, in order to simplify the implementation of the guidelines for all clinicians.
